# The small intestine epithelium exempts Foxp3+ Tregs from their IL-2 requirement for homeostasis and effector function

**DOI:** 10.1172/jci.insight.149656

**Published:** 2021-11-08

**Authors:** Praveen Prakhar, Jaylene Alvarez-DelValle, Hilary Keller, Assiatu Crossman, Xuguang Tai, Yoo Kyoung Park, Jung-Hyun Park

**Affiliations:** 1 Experimental Immunology Branch, Center for Cancer Research, National Cancer Institute, NIH, Bethesda, Maryland, USA.; 2 Department of Surgery, Guthrie Robert Packer Hospital, Sayre, Pennsylvania, USA.; 3 Department of Medical Nutrition-AgeTech-Service Convergence Major, Graduate School of East-West Medical Science, Kyung Hee University, Yongin, South Korea.

**Keywords:** Autoimmunity, Immunology, Adaptive immunity, Cytokines, T cells

## Abstract

Foxp3^+^ Tregs are potent immunosuppressive CD4^+^ T cells that are critical to maintain immune quiescence and prevent autoimmunity. Both the generation and maintenance of Foxp3^+^ Tregs depend on the cytokine IL-2. Hence, the expression of the IL-2 receptor **α**-chain (CD25) is not only considered a specific marker, but also a nonredundant requirement for Tregs. Here, we report that Foxp3^+^ Tregs in the small intestine (SI) epithelium, a critical barrier tissue, are exempt from such an IL-2 requirement, since they had dramatically downregulated CD25 expression, showed minimal STAT5 phosphorylation ex vivo, and were unable to respond to IL-2 in vitro. Nonetheless, SI epithelial Tregs survived and were present at the same frequency as in other lymphoid organs, and they retained potent suppressor function that was associated with high levels of CTLA-4 expression and the production of copious amounts of IL-10. Moreover, adoptive transfer experiments of Foxp3^+^ Tregs revealed that such IL-2–independent survival and effector functions were imposed by the SI epithelial tissue, suggesting that tissue adaptation is a mechanism that tailors the effector function and survival requirements of Foxp3^+^ Tregs specific to the tissue environment.

## Graphical abstract

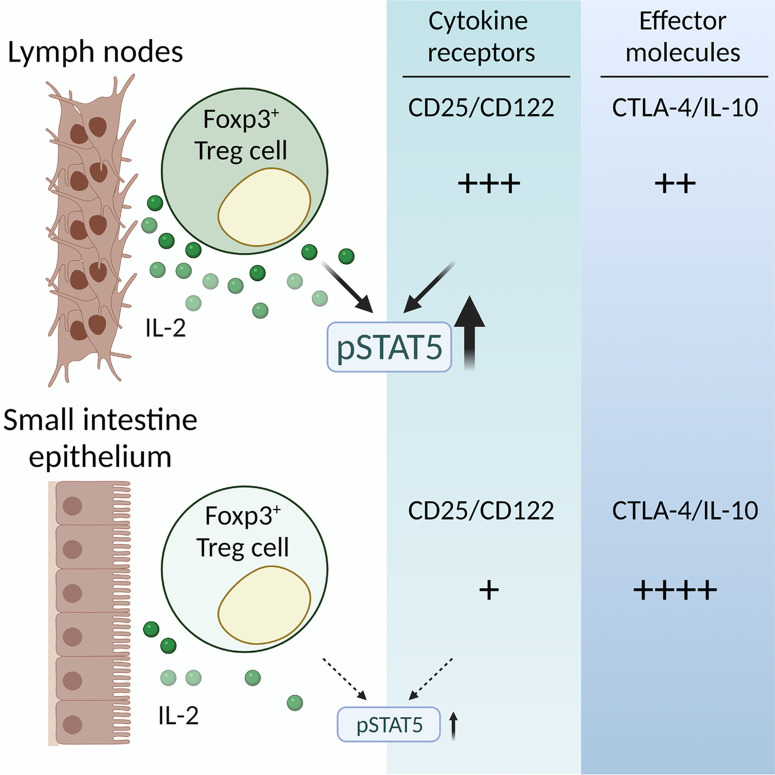

## Introduction

CD4^+^ T cells that express the transcription factor Foxp3 are commonly referred to as Tregs, and such Foxp3^+^ Tregs play nonredundant roles in maintaining the immune quiescence in peripheral tissues (1). Foxp3^+^ Tregs comprise a phenotypically and functionally diverse population, and they express a range of different effector molecules and surface markers, which has led to the identification of multiple Treg subsets (2). Whether these different subsets correspond to terminally differentiated effector cells or whether plasticity remains among the distinct subsets is not fully resolved. To further complicate this issue, Foxp3^+^ Tregs are not only generated in the thymus, but they can also develop from naive CD4^+^ T cells in peripheral organs (3). Thus, the peripheral Foxp3^+^ Treg pool comprises a mixture of cells with different tissue origins and developmental pathways. Molecular markers that can differentiate the developmental history of peripheral Tregs are not yet fully established (2).

A surface marker that is common to most Foxp3^+^ Tregs is the α-chain of the IL-2 receptor (IL-2Rα) (1). The high-affinity IL-2R is composed of IL-2Rα (also referred to as CD25), IL-2Rβ (CD122), and the γc chain (CD132) (4). CD25 plays a critical role in capturing IL-2 and facilitating IL-2R signaling. In resting CD4^+^ T cells, CD25 was found to be associated with Foxp3 expression, so that surface CD25 expression can be employed to identify CD4^+^Foxp3^+^ Tregs (5). Because both the generation and maintenance of Foxp3^+^ Tregs depend on the cytokine IL-2 (6), CD25 is also appreciated as a nonredundant requirement for Foxp3^+^ Treg development. As such, CD25 deficiency results in the failure to produce functional Foxp3^+^ Tregs, and CD25-deficient mice are subject to lethal autoimmunity (7, 8).

Interestingly, the surface expression of CD25 on Foxp3^+^ Tregs is not fixed but displays a substantial degree of variability. Some Foxp3^+^ Tregs in peripheral tissues even fail to express discernible amounts of CD25, and they appear as CD25^lo^ or CD25^–^ Tregs (9). The developmental origin and effector function of CD25^+^ versus CD25^–^Foxp3^+^ Tregs in the periphery remain mostly unexamined. Strikingly, here, we found that Foxp3^+^ Tregs in barrier tissues, specifically in the small intestine (SI) epithelium, were highly enriched for CD25^–^ Tregs. Thus, we aimed to understand the cellular mechanisms that would impose such a phenotype and to further examine the effector phenotype and function of Foxp3^+^ Tregs among intraepithelial lymphocytes (IELs) in the SI.

Unfortunately, we found that the conventional method to enrich IELs, which employs isotonic Percoll gradients (10, 11), was inadequate for the quantitative recovery and analysis Foxp3^+^ Tregs from the SI epithelium. While Foxp3^+^ Tregs correspond to approximately 5% of all lymphocytes in the lymph node (LN), less than 0.5% of SI IELs are Foxp3^+^ Tregs. Thus, Tregs are scarce among SI IELs. Additionally, utilizing Percoll gradients for SI IEL enrichment resulted in a dramatic loss of cell numbers during the isolation process. Consequently, we found it problematic to use the Percoll gradient protocol for the in-depth analysis of SI IEL Tregs. To resolve this issue, here, we established a method to purify IELs without the use of Percoll gradients, and we then employed this protocol to identify and analyze SI IEL Foxp3^+^ Tregs. In brief, based on the observation that EpCAM^+^ intestinal epithelial cells constitute the majority of nonhematopoietic (non-CD45^+^) cells, we depleted EpCAM^+^ cells using a magnetic-activated cell sorting system and documented this method to be highly effective in recovering CD45^+^ hematopoietic cells, including Foxp3^+^ Tregs. Using this method, we demonstrated that the SI environment imposes a unique phenotype on Foxp3^+^ Tregs that is associated with the loss of both CD25 and CD122 but with a substantial increase in CTLA-4 and CD39/CD73 expression. Functional analyses further showed that SI IEL Tregs expressed copious amounts of IL-10 and that they were highly efficient immune suppressors, despite lacking CD25. These results revealed tissue-specific adaptation of Foxp3^+^ Tregs, which has wide-ranging implications for further understanding the role of Tregs in mucosal immunity.

## Results

### Identification and analysis of CD25-disparate Foxp3^+^ Tregs.

**Figure 1:**
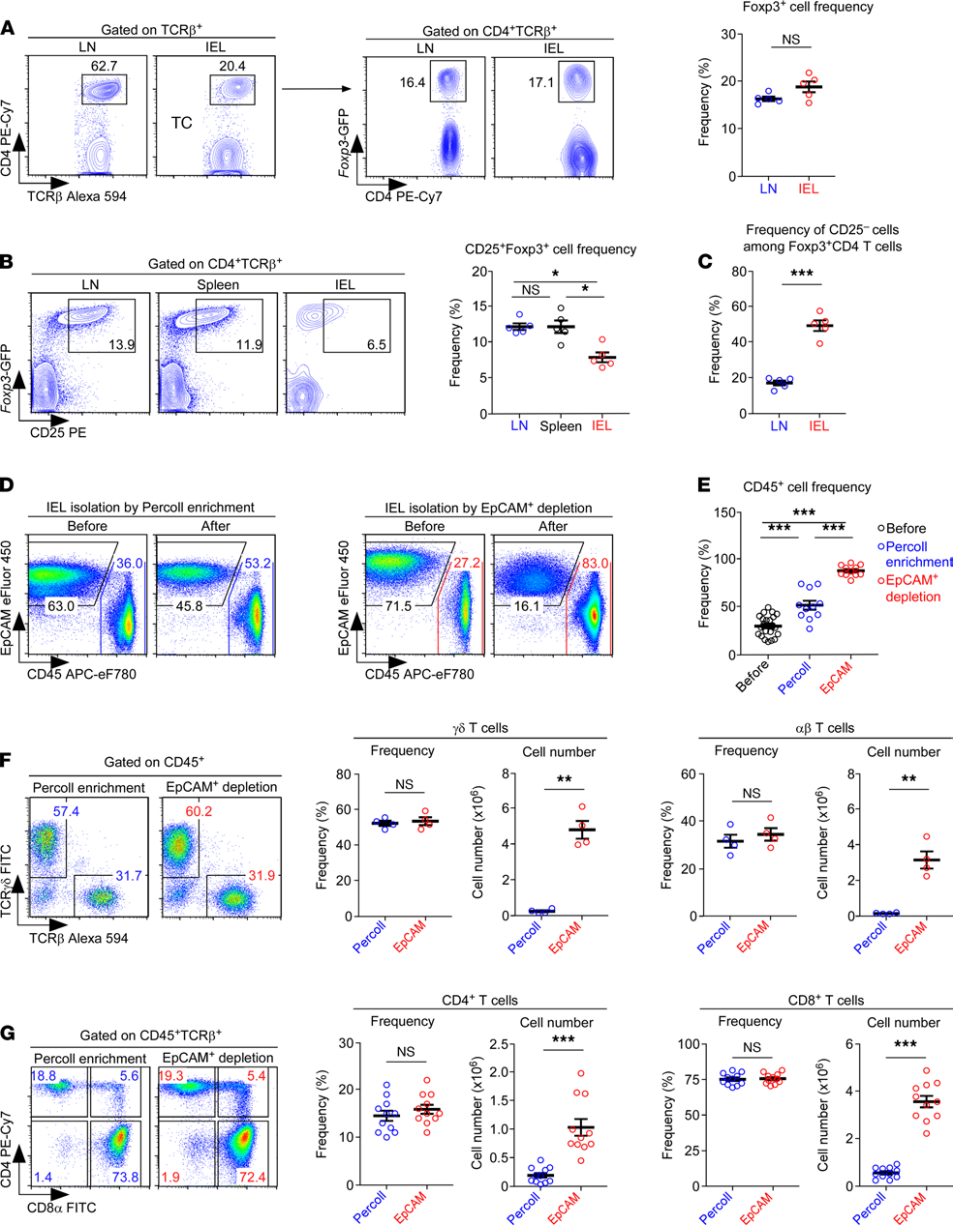
Identification CD25-disparate Foxp3^+^ Tregs and Percoll-free isolation and characterization of SI IELs. (**A**) Identification of Foxp3^+^ Tregs among LN and SI IEL CD4^+^ T cells of *Foxp3*-GFP reporter mice. Foxp3^+^ Treg frequencies were assessed in 2 independent experiments with 5 *Foxp3*-GFP reporter mice. (**B**) Frequencies of CD25^+^Foxp3^+^ Tregs among CD4^+^ T cells in the LN, spleen, and IELs of *Foxp3*-GFP reporter mice. Contour plots (left) are representative, and the graph (right) provides a summary of 2 independent experiments with 5 *Foxp3*-GFP reporter mice. (**C**) Frequencies of CD25^–^ cells among Foxp3^+^CD4^+^ T cells in the LN and IELs of *Foxp3*-GFP reporter mice. The graph provides a summary of 2 independent experiments with 5 *Foxp3*-GFP reporter mice. (**D**) EpCAM versus CD45 expression in SI IELs upon Percoll enrichment (left) or EpCAM^+^ cell depletion (right). The results are representative of 9 independent experiments with a total of 13 WT mice. (**E**) Frequencies of CD45^+^ SI IELs after Percoll enrichment or EpCAM^+^ cell depletion. The graph shows a summary of 9 independent experiments with a total of 11 WT mice. (**F**) γδ and αβ T cell profile of CD45^+^TCRβ^+^ IELs that were isolated by either Percoll enrichment or EpCAM^+^ cell depletion (left). Graph shows the frequency and number of recovered γδ T cells and αβ T cells using the indicated methods (middle and right). The results show a summary of 2 independent experiments with a total of 4 WT mice. (**G**) CD4 and CD8 profiles of CD45^+^TCRβ^+^ IELs that were isolated by either Percoll enrichment or EpCAM^+^ depletion (left). Graphs show the frequency and number of recovered CD4^+^ and CD8^+^ IEL αβ T cells using the indicated methods (middle and right). The results show a summary of 9 independent experiments with a total of 11 WT mice. The data are represented as the mean ± SEM. *P* values were determined by paired Student’s *t* test (**A**, **C**, **F**, and **G**) or 1-way ANOVA with Tukey’s multiple comparison test (**B** and **E**). *P < 0.05, **P < 0.01, ***P < 0.001.

We embarked on this study to understand how the tissue environment shapes the phenotype and function of Foxp3^+^ Tregs. Utilizing *Foxp3*-GFP knock-in reporter mice (12), we first defined Foxp3^+^ Tregs as CD4^+^ T cells that express *Foxp3*-GFP reporter proteins (Figure 1A). Using this criterion, we found that the frequency of Foxp3^+^CD4^+^ T cells did not significantly differ between LN and IELs (Figure 1A). However, the surface expression of CD25, which is associated with terminal differentiation of Foxp3^+^ Tregs (7, 13), significantly varied depending on the tissue environment. As such, the frequency of CD25^+^Foxp3^+^ Tregs was substantially reduced in IELs compared with Tregs in the LN or spleen (Figure 1B). Because the overall frequency of Foxp3^+^ Tregs was not different between the LN and IEL populations (Figure 1A), these results further indicated that IEL CD4^+^ T cells are enriched for Tregs that lack CD25 — i.e., CD25^–^Foxp3^+^ Tregs (Figure 1C). This contrasted with Tregs in the LN and spleen, which were mostly composed of CD25^+^Foxp3^+^ cells (Figure 1B). Thus, the Treg phenotype in the IEL population significantly differed from those in the LN and spleen, suggesting potential tissue-specific adaptation and phenotypic conversion of Foxp3^+^ Tregs.

CD25 is a subunit of the high-affinity IL-2R, whose expression is necessary for efficient IL-2 signaling (4). Because Tregs depend on IL-2 for survival and differentiation (6), the distinct CD25 expression pattern in CD25^–^ versus CD25^+^Foxp3^+^ Tregs could have functional consequences. Therefore, we found it important to examine whether SI IEL Tregs lack IL-2 signaling and, if so, how this deficit would affect their survival and effector function.

### SI IEL enrichment by EpCAM^+^ cell depletion.

A prerequisite for the successful analysis of SI IEL Tregs is their quantitative isolation from the SI epithelium (14). Unfortunately, the conventional protocol of using isotonic Percoll gradients for IEL isolation is plagued by low efficiencies in cell recovery (10, 11). In fact, we found this method being highly inefficient and detrimental for cell viability so that it resulted in a substantial loss of IELs during the isolation process (Supplemental Figure 1A; supplemental material available online with this article; https://doi.org/10.1172/jci.insight.149656DS1). Analyzing the cells obtained by Percoll gradients further revealed that a large fraction of IEL lymphocytes remained in the upper phase of the gradient and failed to accumulate in the interphase (Supplemental Figure 1A). Thus, the Percoll gradient method is suboptimal in recovering and analyzing low-frequency cell populations, such as Foxp3^+^CD4^+^ T cells, which corresponded to less than 0.5% of cells among all CD45^+^ IELs (Supplemental Figure 1B) and less than 1.5% of cells among all CD45^+^TCRβ^+^ IELs (Supplemental Figure 1C). Moreover, the repeated centrifugation steps to enrich the IEL population and to wash out Percoll were highly time consuming and damaging to the cells, thus exacerbating the inefficient cell recovery of the density gradient method. Consequently, we found it necessary to develop an alternative approach for the detailed analysis of SI IEL Foxp3^+^ Tregs.

The lavage of the SI epithelium gives rise to 2 distinct cell populations with different cell sizes (Supplemental Figure 2A, top). The smaller cells (FSC^lo^) corresponded to CD45^+^ lymphocytes (Supplemental Figure 2A, bottom left), whereas the larger cells (FSC^hi^) were mostly intestinal epithelial cells that are EpCAM^+^ (Supplemental Figure 2A, bottom right). Notably, processing the SI epithelial lavage samples through Percoll gradients poorly separated intestinal epithelial cells from IELs (Supplemental Figure 2B, left). Hence, as a superior alternative, we developed a Percoll-free method based on the depletion of epithelial cells using anti-EpCAM antibodies (15) and anti-IgG antibody–conjugated magnetic beads. EpCAM is a transmembrane glycoprotein that is primarily expressed on epithelial cells and absent on CD45^+^ cells of hematopoietic origin (16). We found that the depletion of EpCAM^+^ cells vastly improved the isolation of CD45^+^ cells compared with the conventional Percoll gradient method (Figure 1D and Supplemental Figure 2B, right). Indeed, the postpurification frequency of CD45^+^ cells was significantly higher for the EpCAM^+^ cell depletion method than for the Percoll gradient method (Figure 1E). Moreover, when we assessed the rate of cell recovery, which we determined by dividing the number of CD45^+^ cells after enrichment by the number of CD45^+^ cells before enrichment, we realized that the Percoll gradient method recovered only 15% of the estimated lymphocytes in the starting cell suspension (Supplemental Figure 2C). These results indicated that approximately 85% of the lymphocytes were lost during the enrichment process when using the Percoll gradient method. In marked contrast, the EpCAM^+^ cell depletion method recovered close to 90% of the lymphocytes in the initial SI cell emulsion (Supplemental Figure 2C), demonstrating that the EpCAM^+^ cell depletion method is highly efficient and results in minimal loss of IELs during the purification process.

### Phenotypic and functional analyses of SI IEL T cells.

Next, we aimed to confirm that the cellular composition and phenotype of IELs recovered by EpCAM^+^ depletion did not differ from those isolated by Percoll gradients. Indeed, the frequencies of γδ T cells and αβ T cells among SI IELs did not differ between the 2 isolation procedures (Figure 1F). However, the total numbers of recovered γδ and αβ T cells dramatically increased when isolated by EpCAM^+^ depletion (Figure 1F). Consequently, the numbers but not the frequencies of CD4^+^ and CD8^+^ T cells obtained by anti-EpCAM isolation were substantially increased (Figure 1G). We also considered it important to demonstrate that EpCAM^+^ cell depletion did not adversely affect the function of purified IELs. To this end, we stimulated EpCAM-depleted and Percoll-isolated SI IELs with plate-bound anti-CD3 antibodies and a cocktail of γc family cytokines — i.e., IL-2, IL-7, and IL-15 — to assess proinflammatory cytokine production after 5 days of cell culture (17). Gating on CD8^+^ αβ T cells, we found that the IEL T cells isolated with either of the isolation methods had successfully differentiated into effector T cells, producing copious amounts of IFN-γ and TNF-α upon PMA and ionomycin stimulation (Supplemental Figure 3). Altogether, these results indicated that the EpCAM^+^ depletion method is superior to the conventional Percoll gradient method, as it permits the efficient and quantitative recovery of T cells from SI epithelial tissues while preserving their function.

### Phenotypic analysis of Foxp3^+^ Tregs in the epithelium of the SI.

**Figure 2:**
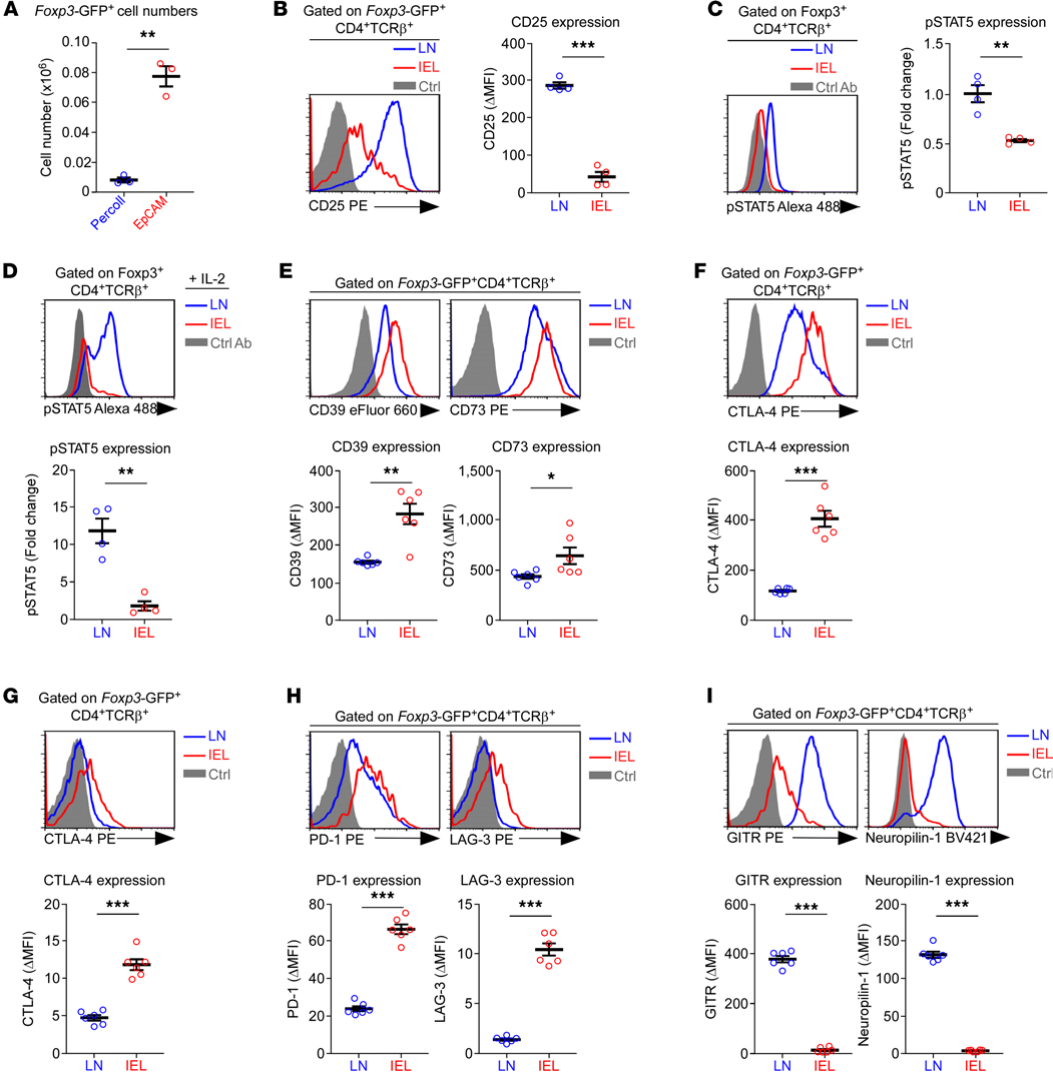
Phenotypic and functional characterization of SI IEL Foxp3^+^ Tregs. (**A**) Cell numbers of SI IEL Foxp3^+^ Tregs isolated from *Foxp3*-GFP reporter mice by Percoll enrichment or by EpCAM^+^ cell depletion. The graph shows the summary of 2 independent experiments with a total of 3 *Foxp3*-GFP reporter mice. (**B**) Surface expression (left) and ΔMFI (right) of CD25 on *Foxp3*-GFP^+^CD4^+^TCRβ^+^ cells from LN and IELs. The histogram is representative, and the graph shows the summary of 2 independent experiments with a total of 4 *Foxp3*-GFP reporter mice. (**C**) Intracellular pSTAT5 in freshly isolated Foxp3^+^ Tregs of LN and SI IELs. The histogram is representative (left), and the graph is a summary (right), of 3 independent experiments with a total of 4 WT mice. (**D**) IL-2–induced STAT5 phosphorylation in Foxp3^+^ Tregs of LN and SI IELs. Freshly isolated lymphocytes of LN and SI IELs were stimulated with recombinant IL-2 (1 ng/mL) for 30 minutes and then assessed for pSTAT5 contents gated on Foxp3^+^ Tregs. Histogram is representative (top), and the fold increase in pSTAT5 (bottom graph) is the summary, of 2 independent experiments with a total of 4 WT mice. (**E**) Surface expression (top) and ΔMFI (bottom) of CD39 and CD73 on *Foxp3*-GFP^+^CD4^+^ T cells from LN and IELs. Histograms are representative (top), and the graphs show the summary (bottom), of 2 independent experiments with a total of 6 *Foxp3*-GFP reporter mice. (**F**) Intracellular staining for CTLA-4 in *Foxp3-*GFP^+^CD4^+^ T cells from LN and IELs. Histograms are representative (top), and the ΔMFI graphs show the summary (bottom), from 2 independent experiments with a total of 6 *Foxp3*-GFP reporter mice. (**G**) Surface expression of CTLA-4 on *Foxp3*-GFP^+^CD4^+^ T cells from LN and IELs. Histogram is representative (top), and the ΔMFI graph shows the summary (bottom), from 2 independent experiments with a total of 6 *Foxp3*-GFP reporter mice. (**H**) Surface expression of PD-1 and LAG-3 on *Foxp3*-GFP^+^CD4^+^ T cells from LN and IELs. Histograms are representative (top), and the ΔMFI graphs show the summary (bottom), from 2 independent experiments with a total of 6 *Foxp3*-GFP reporter mice. (**I**) Surface expression (top) and ΔMFI (bottom) of GITR and Neuropilin-1 on *Foxp3*-GFP^+^CD4^+^ T cells from LN and IELs. Histograms are representative (top), and the graph show the summary (bottom), of 2 independent experiments with a total of 6 *Foxp3*-GFP reporter mice. The data are represented as the mean ± SEM. *P* values were determined by paired Student’s *t* test. *P < 0.05, **P < 0.01, and ***P < 0.001.

Having established an efficient protocol for T cell isolation from the SI epithelium, we next asked whether the EpCAM^+^ cell depletion method would be also applicable to recover SI IEL Foxp3^+^ Tregs. Indeed, the number of recovered Foxp3^+^ Tregs dramatically increased (8- to 10-fold) when using the EpCAM^+^ depletion method compared with the Percoll enrichment method (Figure 2A), even as the frequency of Foxp3^+^ Tregs remained comparable (Supplemental Figure 4A). Thus, EpCAM^+^ depletion permits effective and quantitative recovery of Foxp3^+^ cells from SI epithelial tissues, facilitating the in-depth analysis of SI IEL Tregs at high resolution.

Equipped with this tool, we assessed the phenotype of SI IEL Foxp3^+^ Tregs with the goal of identifying tissue-specific characteristics distinct to Tregs in the SI epithelium. As shown above (Figure 1B), the most striking feature of SI IEL Foxp3^+^ Tregs was their dramatic loss of CD25 expression compared with Foxp3^+^ Tregs in the LN (Figure 2B). CD25 is a hallmark of Foxp3^+^ cells and is commonly used to identify mature Foxp3^+^ Tregs (5, 18, 19). CD25 expression is also considered a nonredundant requirement for Foxp3^+^ Treg generation and homeostasis, which raises the issue of how SI IEL Tregs would bind IL-2 and transduce IL-2 signaling without significant amounts of surface CD25 (Figure 2B). Moreover, IL-2 signaling upregulates the expression of CD122; hence, the relative abundance of CD122 can serve as a marker to indicate the amount of IL-2 signaling in a given cell (18, 19). In this regard, it was revealing that SI IEL Foxp3^+^ Tregs expressed significantly lower amounts of CD122 than LN Foxp3^+^ Tregs, suggesting diminished IL-2 signaling in vivo (Supplemental Figure 4B).

To directly demonstrate that SI IEL Foxp3^+^ Tregs are impaired in IL-2 signaling, we next assessed the intracellular phospho-STAT5 (pSTAT5) contents of Foxp3^+^ Tregs, immediately after their isolation from the SI epithelium or LNs. We previously established an effective fixation/staining protocol for the simultaneous detection of Foxp3 and pSTAT5 (20, 21). Here, we employed this approach to document that SI IEL Foxp3^+^ Tregs contained significantly less pSTAT5 than their LN counterpart ex vivo (Figure 2C). Along these lines, SI IEL Foxp3^+^ Tregs were also severely impaired in their IL-2 responsiveness, as illustrated by their inability to induce STAT5 phosphorylation upon in vitro IL-2 stimulation (Figure 2D). Collectively, these results indicated that Foxp3^+^ Tregs in the SI epithelium lack IL-2 signaling in situ.

Because IL-2 signaling is important to induce and maintain Foxp3^+^ Treg functions (7), these results further suggested that the expression of Treg effector molecules could have been adversely affected (22). Surprisingly, however, this was not the case. SI IEL Foxp3^+^ Tregs abundantly expressed both CD39 and CD73, two critical surface molecules that mediate Treg suppressor function (Figure 2E) (23). CD39 and CD73 are ectoenzymes that hydrolyze extracellular ATP (eATP) to AMP and, ultimately, to adenosine, an immunosuppressive nucleoside for T cells (24). Additionally, the expression of CTLA-4, a critical negative immunoregulator (25), was highly elevated in SI IEL Foxp3^+^ Tregs (Figure 2F). In fact, the amount of CTLA-4 was induced to such high levels that we were able to detect CTLA-4 expression on the cell surface of Foxp3^+^ Tregs (Figure 2G). The presence of surface CTLA-4 is an unusual phenomenon because CTLA-4 proteins are constitutively internalized so that they are normally only found intracellularly (26). In addition to CTLA-4, the expression of other immunosuppressive molecules, such as PD-1 (27) and LAG-3 (28), was also markedly increased in SI IEL Foxp3^+^ Tregs (Figure 2H). On the other hand, not all effector molecules were increased in their abundance because GITR and Neuropilin-1 expression was dramatically reduced in the same cells (Figure 2I) (23, 29). These results indicated that the Treg effector phenotype associated with the SI epithelium is due to an overall increase in surface protein expression but is limited to a select set of effector molecules. Altogether, SI IEL Tregs displayed a highly activated phenotype, which we found surprising because they lacked IL-2 signaling.

### Functional analysis of Foxp3^+^ Tregs in the SI epithelium.

**Figure 3:**
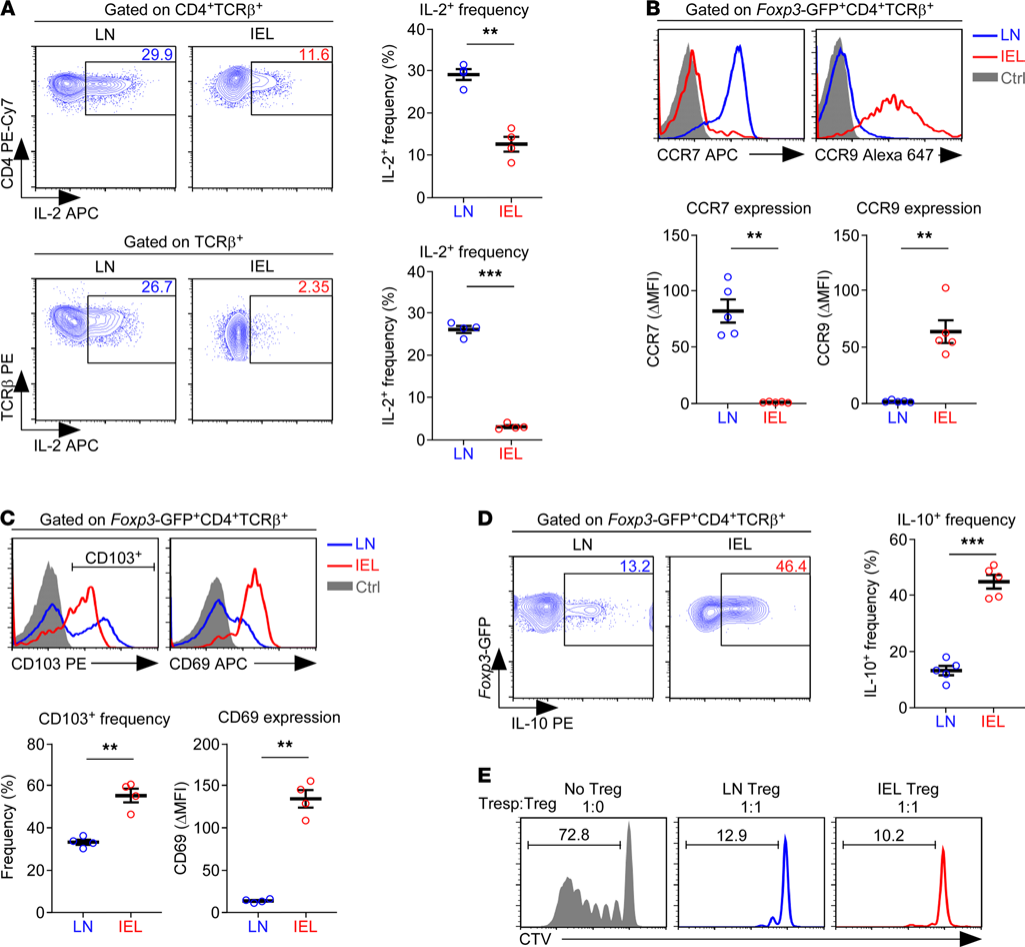
SI IEL-associated features of Foxp3^+^ Tregs. (**A**) IL-2 expression in LN and IEL CD4^+^ (top) and total T cells (bottom) in LN and IEL populations. Contour plots show staining for intracellular IL-2 in CD4^+^ and total T cells from the indicated organs upon PMA and ionomycin stimulation. The graph shows the summary of 2 independent experiments with 4 WT mice. (**B**) Histograms show CCR7 and CCR9 expression on *Foxp3*-GFP^+^CD4^+^ T cells from LN and IELs (top). Graphs show the ΔMFI of CCR7 and CCR9 expression (bottom). Histograms are representative, and the graphs show a summary of 2 independent experiments with a total of 5 *Foxp3*-GFP reporter mice. (**C**) Histograms show CD103 and CD69 expression on *Foxp3*-GFP^+^CD4^+^ T cells LN and IELs (top). The graphs show the frequency of CD103^+^ cells and the ΔMFI of CD69 expression (bottom). Histograms are representative, and the graphs show a summary of 2 independent experiments with a total of 4 *Foxp3*-GFP reporter mice. (**D**) IL-10 expression in LN and IEL Foxp3^+^ Tregs. Contour plots show staining for intracellular IL-10 in Foxp3^+^ Tregs from the indicated organs upon PMA + ionomycin stimulation (left). The graph shows the summary of 3 independent experiments with 5 *Foxp3*-GFP reporter mice (right). (**E**) Treg suppression assays with FACS-sorted Foxp3^+^ Tregs from LN and IELs of *Foxp3*-GFP reporter mice. Cell Trace Violet (CTV) dilution was monitored to visualize the proliferation of responder T cells (Tresp) after 5 days of in vitro stimulation in the absence or presence of Tregs at a 1:1 ratio. Data are representative of 2 independent experiments. The data are represented as the mean ± SEM. *P* values were determined by paired Student’s *t* test. **P < 0.01, and ***P < 0.001.

To understand whether the impaired IL-2 signaling in SI IEL Foxp3^+^ Tregs was entirely a cell-intrinsic problem or if environmental factors also play a role, we next assessed the IL-2 availability for SI IELs. IL-2 is a T cell–derived cytokine that is primarily produced by activated CD4^+^ T cells and, to a lesser extent, by CD8^+^ T cells (30). Thus, we assessed IL-2 production of PMA and ionomycin-stimulated CD4^+^ T cells from the LN and IELs, and we found that the frequency of IL-2–expressing CD4^+^ T cells was dramatically decreased in the IELs compared with LN cells (Figure 3A, top). Moreover, the IL-2^+^ cell frequency among all αβ T cells in the IEL population was even more reduced (Figure 3A, bottom), indicating that the overall IL-2 availability in the SI epithelium is very low. Additionally, the IL-2 mRNA content in CD44^hi^ effector T cells, which comprise the IL-2–producing cells under steady-state condition, was significantly reduced in IEL T cells compared with their LN counterparts (Supplemental Figure 4C, top). IFN-γ mRNA expression, on the other hand, was highly elevated in IEL T cells, indicating that the downregulation of cytokine expression in IEL T cells is specific to IL-2 (Supplemental Figure 4C, bottom). These results suggested that the lack of IL-2 signaling in SI IEL Foxp3^+^ Tregs is the result of the compound effect of diminished IL-2 production by SI IEL T cells and the low amount of IL-2R — i.e., CD25 and CD122 — expression on SI IEL Foxp3^+^ Tregs themselves.

SI IEL T cells are mostly tissue resident. Thus, they lack surface molecules that would drive them into the circulation, but they abundantly express surface proteins that retain them in the tissue (31). To examine whether SI IEL Tregs are tissue resident or whether they correspond to newly immigrated Tregs, we assessed the expression of the chemokine receptors CCR7 and CCR9. While CCR7 drives the migration of T cells into lymphoid organs, CCR9 is required for T cell recruitment into the SI (32). We identified a reciprocal relationship between CCR7 and CCR9 expression in Foxp3^+^ Tregs of the LN and SI epithelium; LN Tregs expressed high levels of CCR7 and low amounts of CCR9, while SI IEL Tregs expressed high levels of CCR9 and low amounts of CCR7 (Figure 3B). Thus, SI IEL Foxp3^+^ Tregs are distinct from LN Foxp3^+^ Tregs in their tissue-tropic chemokine receptors. Consistent with these features, we were able to locate them in the villi of the SI epithelial tissue where other tissue-resident T cells reside (Supplemental Figure 4D). In fact, assessing the phenotypic markers of tissue residency revealed that most Foxp3^+^ Tregs in the SI IEL population expressed the tissue-retention molecule CD103 (33) and that they also expressed large amounts of CD69, a C-type lectin that impedes migration into the circulation (Figure 3C) (27). In contrast, many LN Foxp3^+^ Tregs lacked CD103 expression, and the amount of CD69 expression was also markedly reduced, with most LN Tregs lacking CD69 expression (Figure 3C). CD62L is another important cell adhesion molecule that facilitates the entry of naive T cells into lymphoid tissues (34). While the expression of the activation/memory marker CD44 did not significantly differ between SI IEL and LN Foxp3^+^ Tregs, CD62L was absent on SI IEL Foxp3^+^ Tregs (Supplemental Figure 5A). Thus, SI IEL Foxp3^+^ Tregs differed from their LN counterparts in that they displayed surface markers reminiscent of tissue-resident memory T cells (31).

Having established that SI IEL Tregs display markers of tissue residency, we next asked whether they are functional and, thus, can contribute to maintaining immune quiescence in the SI epithelium. To this end, we examined cytokine production in SI IEL Tregs and found them to be highly effective producers of the immunosuppressive cytokine IL-10 (Figure 3D). In fact, the frequency of IL-10 producers among SI IEL Tregs vastly exceeded that among LN Tregs (Figure 3D). To directly assess whether such a strong effector phenotype would translate into suppressive activity, we next sorted Foxp3^+^ Tregs by FACS from the LN and SI IEL of *Foxp3*-GFP reporter mice and set up Treg suppression assays (35). These assays documented that both SI IEL and LN Tregs potently suppressed the in vitro proliferation of responder T cells (Tresp), confirming the suppressor function of SI IEL Tregs (Figure 3E and Supplemental Figure 5B). These results correlated the suppressor activity of the Tregs with the expression of effector molecules, such as CD39/CD73, CTLA-4, and IL-10. However, these data also suggest that CD25 expression — at least in SI IEL Tregs — is not required for immune suppression or the effector function of Foxp3^+^ Tregs.

### Tissue adaptation of Foxp3^+^ Tregs in the SI epithelium.

**Figure 4:**
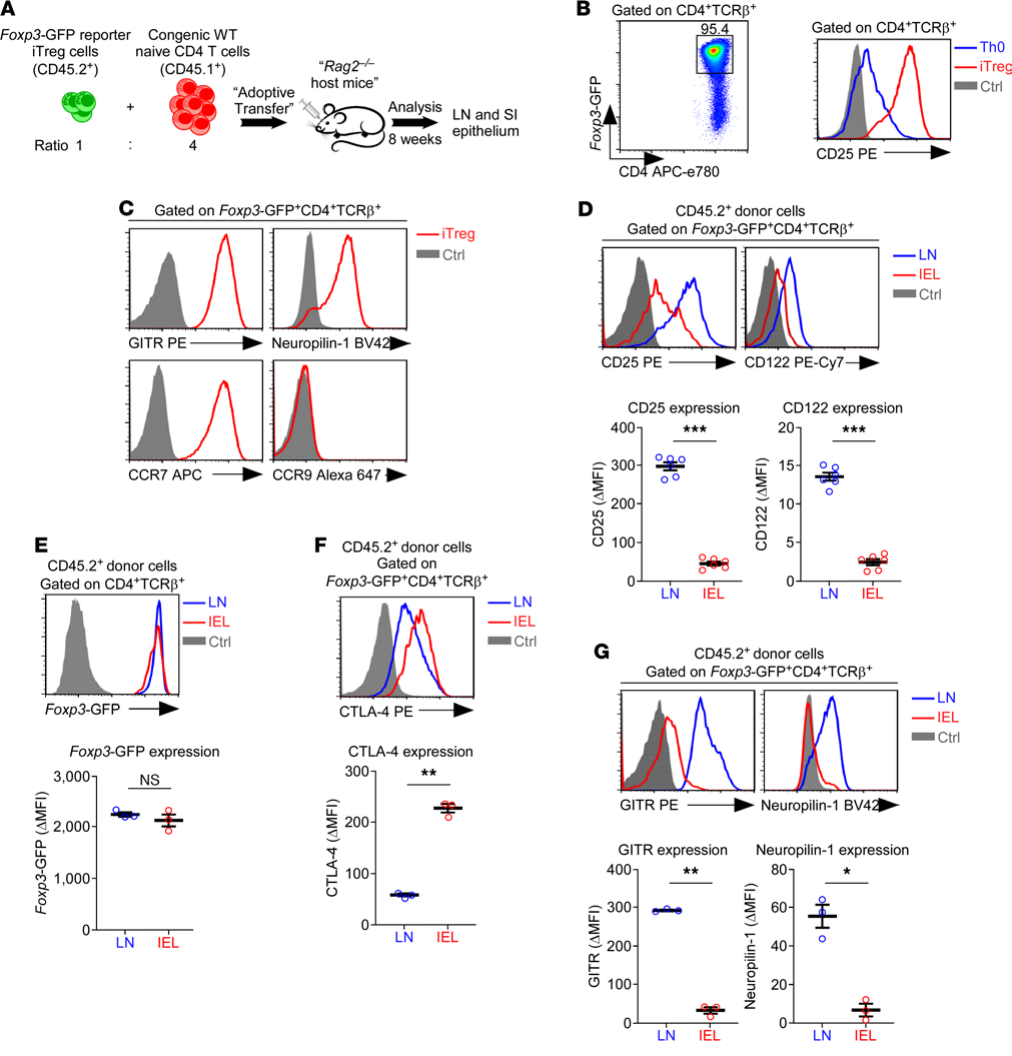
Tissue adaptation of adoptively transferred Foxp3^+^ Tregs in SI IELs. (**A**) Adoptive transfer of Foxp3^+^ Tregs. Naive CD4^+^ T cells from *Foxp3*-GFP reporter mice (CD45.2^+^) were induced to differentiate into Foxp3^+^ Tregs in vitro and coinjected with freshly purified naive CD4^+^ T cells from congenic mice at a 1:4 ratio into *Rag2*-deficient recipient mice. Eight weeks after transfer, donor T cells were recovered from the LN and SI epithelium of host mice and assessed for Treg phenotypes after gating based on the CD45.2^+^ congenic marker. (**B**) *Foxp3*-GFP and CD25 expression was assessed in in vitro–differentiated naive CD4^+^ T cells stimulated with anti-TCR antibodies in the presence of IL-2 and TGF-β. The results are representative of 3 independent experiments. (**C**) In vitro–generated Foxp3^+^ iTregs were assessed for the expression of the indicated surface molecules. The results are representative of 2 independent experiments. (**D**) Histograms show CD25 and CD122 expression on *Foxp3*-GFP^+^ donor T cells from recipient LN and IEL populations (top). Graphs show the ΔMFI of CD25 and CD122 expression (bottom). The histograms are representative, and the graphs show the summary of 3 adoptive transfer experiments with a total of 6 host mice. (**E**) *Foxp3*-GFP reporter expression (top) and ΔMFI of *Foxp3*-GFP (bottom) in donor *Foxp3*-GFP^+^CD4^+^ Tregs from LN and IELs of *Rag2*^–/–^ recipient mice. The histogram is representative, and the graph shows a summary of 3 adoptively transferred host mice. (**F**) Intracellular staining for CTLA-4 (top) and ΔMFI of CTLA-4 expression (bottom) in *Foxp3*-GFP+CD4+ donor T cells from LN and IELs of *Rag2–/–* recipient mice. The histogram is representative, and the graph shows the summary from 3 adoptive transfer experiments. (**G**) Surface staining for GITR and Neuropilin-1 (top) and ΔMFI of GITR and Neuropilin-1 expression (bottom) in *Foxp3*-GFP+CD4+ donor T cells from LN and IELs of *Rag2–/–* recipient mice. The histogram is representative, and the graph shows the summary from 3 adoptive transfer experiments. The data are represented as the mean ± SEM. *P* values were determined by paired Student’s *t* test. *P < 0.05, **P < 0.01, ***P < 0.001.

Because of the difference in CD25 expression among Tregs (Figure 1B), we next asked whether the loss of CD25 on SI IEL Foxp3^+^ Tregs is an acquired trait. To this end, we designed an experiment in which Foxp3^+^ Tregs were adoptively transferred into lymphopenic recipient mice and then monitored for their phenotypic differentiation in the LN versus the SI epithelium (Figure 4A). First, we isolated naive CD4^+^ T cells from the LNs of *Foxp3*-GFP reporter mice and then induced them to differentiate into CD25^+^Foxp3^+^ induced Tregs (iTregs) in vitro by TCR stimulation in the presence of IL-2 and TGF-β (Figure 4B) (35, 36). Phenotyping the in vitro–generated Foxp3^+^ Tregs revealed that such iTregs expressed large amounts of the chemokine receptor CCR7 but minimal amounts of CCR9 (Figure 4C). On the other hand, GITR and Neuropilin-1 expression was highly elevated in the same cells (Figure 4C). Altogether, these results suggested that in vitro–generated iTregs phenotypically corresponded to Foxp3^+^ Tregs in lymphoid organs and that they differed from IEL Foxp3^+^ Tregs (Figure 3B and Figure 2I). Therefore, we considered it appropriate to employ iTregs as donor cells to determine whether the SI epithelial environment would impose gut-specific features on Foxp3^+^ Tregs. To do so, we sorted mature Foxp3^+^ Tregs by FACS from in vitro–generated iTregs based on their CD25 and *Foxp3*-GFP reporter expression (CD25^+^*Foxp3*-GFP^+^) and then coinjected the sorted iTregs with freshly isolated congenic (CD45.1) naive CD4^+^ T cells at a 1:4 ratio into *Rag2*-deficient lymphopenic recipient mice (Figure 4A). Foxp3^+^ Tregs in lymphoid tissues depend on IL-2, but they do not produce IL-2 themselves; therefore, Tregs require exogenous IL-2 for their survival and homeostasis (37). Consequently, we coinjected congenic bystander CD4^+^ T cells as the source of exogenous IL-2 so that they may supply IL-2 for the homeostatic maintenance of donor Foxp3^+^ Tregs. Eight weeks after the transfer, we recovered donor T cells from the LNs and SI, and we assessed the phenotypic differences in *Foxp3*-GFP^+^ reporter donor T cells between these organs (Supplemental Figure 6A).

Strikingly, the CD25^+^*Foxp3*-GFP^+^ donor cells displayed dramatic differences in their IL-2R expression depending on their site of migration (Figure 4D). SI IEL Foxp3^+^ Tregs expressed substantially diminished levels of both CD25 and CD122 compared with their LN counterparts (Figure 4D). Such difference in IL-2R expression phenocopied the situation under steady-state conditions in LN and SI IELs of WT mice, suggesting that the tissue environment imposes the distinct surface cytokine receptor expression patterns on Foxp3^+^ Tregs. Unlike the IL-2R, however, the amount of Foxp3 proteins was comparable between LN and SI IEL Foxp3^+^ Tregs (Figure 4E), which was unexpected because IL-2 signaling is considered critical to upregulate and maintain Foxp3 expression. Moreover, CTLA-4 expression was also highly induced in Foxp3^+^ donor cells that had migrated into the SI epithelium compared with those residing in the LN (Figure 4F). Such upregulation in expression was specific to CTLA-4, because the amount of GITR and Neuropilin-1 on Tregs (29) was decreased upon taking up residency in the SI epithelium (Figure 4G), which was accompanied with an increase in CCR9 expression (Supplemental Figure 6B). Collectively, these results indicated that Foxp3^+^ Tregs in the SI epithelium maintain Foxp3 expression and reside in this tissue in an IL-2–independent fashion and that such an ability is endowed by the SI tissue environment.

## Discussion

Foxp3^+^ Tregs utilize multiple mechanisms to maintain immune quiescence in peripheral tissues (23). Thus, it would be reasonable to assume that Tregs employ different tools to confer immune suppression, depending on the cellular context and in a tissue-specific manner. Here, we report that Foxp3^+^ Tregs undergo tissue-dependent adaptation so that Tregs in the SI epithelium displayed dramatically diminished IL-2R expression and lacked GITR and Neuropilin-1. At the same time, SI epithelial Foxp3^+^ Tregs expressed high levels of CD39/CD73 and CTLA-4, and they produced copious amounts of IL-10 compared with Foxp3^+^ Tregs in the LN. Despite such differences, SI IEL Tregs remained equipotent to LN Foxp3^+^ Tregs in suppressing Tresp proliferation. Thus, tissue adaptation is a mechanism that tailors the effector phenotype of Foxp3^+^ Tregs according to the tissue environment.

Foxp3^+^ Tregs are composed of different subtypes. While all Tregs express the transcription factor Foxp3, they can be divided into distinct subsets depending on their developmental origin, effector function, and phenotype (38). As such, there are thymic Tregs (tTregs) or natural Tregs (nTregs) that develop in the thymus, peripheral Tregs (pTregs) that are generated in the periphery, eTregs that are Tregs with potent effector function, and iTregs that are induced from naive CD4^+^ T cells in vitro to become Tregs (39). Multiple molecules have been proposed to distinguish these Treg subsets, such as Helios, Neuropilin-1, CD44, and CD103 (39). Whether their expression is the cause or consequence of subtype-specific differentiation remains disputed and represents a field of active research. Another pending question on Treg subset differentiation is whether these different Treg subtypes represent terminally differentiated cells or whether they remain plastic and can convert or revert into different phenotypes with distinct effector functions. Tregs can display context-dependent differences in their phenotypes, which has been demonstrated using T-bet, GATA-3, and Foxp3 tricolor-reporter mice (40). Here, it was found that GATA-3^+^ Tregs can be converted into T-bet^+^ Tregs and vice versa via cytokines produced under mild inflammatory conditions (40). Thus, the permeation of environmental factors, such as cytokines, can alter the transcriptional circuitry of Tregs. Our current study suggests that the tissue environment of the gut mucosa also represents such a circumstance where the phenotype and function of Tregs can be altered, and these results further bolster the view that peripheral Foxp3^+^ Tregs possess substantial plasticity.

The cellular mechanisms that control the abundance and effector functions of SI IEL Tregs are poorly understood. Intestinal Foxp3^+^ Tregs can expand in situ by gut-associated DCs or intestinal epithelial cells in an MHC-II–dependent manner (41). Alternatively, Foxp3^+^ Tregs outside of the gut can be recruited and retained in the SI epithelium in a CD103-dependent fashion (42). In the current study, we employed an adoptive transfer model of Foxp3^+^ iTregs to assess their phenotypic conversion in the gut. Notably, the recruitment of donor iTregs and the establishment of tissue residency in the SI epithelium were less efficient than in the LN or spleen. A potential explanation for the delayed iTreg recruitment to the SI epithelium could be their lack of the gut homing receptor CCR9, which is normally not expressed on CD4-lineage T cells but is required for T cell migration into intestinal tissues (43). Along these lines, we documented that in vitro–generated iTregs did not express CCR9. Whether the forced expression of CCR9 would resolve this issue and expedite the migration of Foxp3^+^ iTregs into the SI epithelium is an interesting possibility that remains to be assessed. Addressing this issue would help improve our understanding of the tissue origin of SI IEL Tregs under steady-state conditions. We recently generated CCR9-transgenic mice that express a mouse *Ccr9* cDNA under the control of the human *CD2* promoter/enhancer, and we aim to address this question using these mice in future studies.

Cytokines, specifically IL-2, play a critical role in the generation and maintenance of Foxp3^+^ Tregs (44). While IL-2 is considered a nonredundant requirement for the thymic development of Tregs, it has been acknowledged that cytokines other than IL-2 can maintain the survival of peripheral Tregs (45, 46). In this regard, we found it interesting that SI IEL Foxp3^+^ Tregs expressed substantially lower amounts of CD122 and CD25 than their counterparts in lymphoid tissues, which resulted in impaired binding and signaling of IL-2. Therefore, the survival of Tregs in the SI epithelium is likely independent of IL-2 and potentially dependent on other cytokines. IL-15 is a prominent homeostatic cytokine that is highly expressed in intestinal tissues (47). However, we consider it unlikely that IL-15 maintains the survival of SI IEL Tregs. IL-15 requires CD122 for signaling, but CD122 expression is dramatically downregulated on these cells. Therefore, SI IEL Tregs probably utilize CD122-independent cytokines to support their survival in the intestine. We propose that such a conversion of survival cytokine utilization is imposed by the SI epithelial tissue. The biological significance of this event still needs to be determined. The prosurvival cytokine for SI IEL Tregs also remains to be identified. As such, it has been previously documented that IL-7 can replace IL-2 signaling in the survival of peripheral Tregs and that IL-7 can contribute to the peripheral homeostasis of Tregs (48). Adoptive transfer of iTregs into IL-7–deficient *Rag2*^–/–^ recipient mice could be an experiment that tests this possibility.

Finally, it is unclear what specific signals in the SI epithelium would induce the phenotypic changes associated with SI epithelial Tregs. Here, we wish to point out that changes in cytokine receptor expression and other phenotypic features did not result in the conversion of Foxp3^+^ Tregs into other CD4 effector T cell types. While effector CD4^+^ T cells in peripheral tissues have substantial plasticity (49, 50), the tissue adaptation of Foxp3^+^ Tregs to the SI epithelium is distinct, as their cellular identity as Foxp3^+^ Tregs does not change. Additional studies are required to identify the cellular cues controlling SI epithelium–specific adaptation of iTregs, and these questions will need to be answered in the context of deciphering the molecular mechanisms that establish the subset identity of Foxp3^+^ Tregs.

## Methods

Supplemental methods are available online with this article.

### Mice.

C57BL/6 (B6) and CD45.1 congenic B6 mice were obtained from the Charles River Laboratories. *Rag2*^–/–^ mice were purchased from The Jackson Laboratory. *Foxp3*-GFP–knock-in reporter mice were provided by Vijay K. Kuchroo (Harvard Medical School, Boston, Massachusetts, USA) (12). Unless mentioned otherwise, all experiments were performed using both male and female mice that were 6–12 weeks old at the start of the experiment.

### Flow cytometry.

Single cell suspensions were prepared from the indicated organs as previously described (51) and were stained with fluorophore-conjugated antibodies at 4°C for 30 minutes. For intracellular staining, cells were fixed and permeabilized with Foxp3/Transcription factor staining buffer (eBioscience). Dead cells were gated out by counter staining with Ghost Dye (Tonbo Biosciences) and based on forward scatter profiles. Data were acquired on LSRII, LSR Fortessa, and Fortessa X-20 cytometers (BD Biosciences) and analyzed using FlowJo v10.5.0 software (FlowJo). Antibodies with the following antigen specificities and conjugated with the indicated fluorochrome dyes were used: γδTCR-FITC (GL3, BD Biosciences), CD8α-FITC (53-6.7, BD Biosciences) or CD8α-BV786 (53-6.7, BD Biosciences), Isotype Alexa Fluor 488 (MOPC-21, BD Biosciences), pSTAT5–Alexa Fluor 488 (47/Stat5, BD Biosciences), CD25-PE (PC61.5, eBioscience), TNF-α–PE (MP6-XT22, BioLegend), CD103-PE (2E7, Invitrogen), TCRβ-PE (H57-597, BD Biosciences), CD73-PE (TY/11.8, BioLegend), GITR-PE (DTA-1, eBioscience), CTLA-4-PE (UC10-4F10-11, Tonbo Bioscience), IL-10–PE (JES5-16E3, eBioscience), CD122-PE (TM-β1, BD Biosciences), PD-1–PE (29F.1A12, BioLegend), LAG-3–PE (C9B7W, BioLegend), CD4–PE-Cy7 (GK1.5, Tonbo Bioscience), CD122–PE-Cy7 (TM-β1, Invitrogen), CD45.2–PE-Cy7 (104, Tonbo Biosciences), Neuropilin-1–BV421 (3E12, BioLegend), EpCAM-eFluor450 (G8.8, Invitrogen), CD44-BV421 (IM7, BioLegend), TCRβ-BV421 (H57-597, BioLegend), CD45.2-eFluor450 (104, eBioscience), CCR9-eFluor450 (CW-1.2, Invitrogen), CD8β–Pacific Blue (53-.5.8, BioLegend), TCRβ–Alexa Fluor 594 (H57-597, BioLegend), CD25–Alexa Fluor 594 (PC61, BioLegend), CD69-APC (H1.2F3, BioLegend), IFN-γ–APC (XMG1.2, BioLegend), CD62L-APC (MEL-14, eBioscience), CD45.1-APC (A20, BioLegend), CD8α–Alexa Fluor 647 (53-6.7, BioLegend), CD39-eFluor660 (eBio1D3, eBioscience), Foxp3-eFluor660 (FJK-16s, Invitrogen), CCR7-APC (4B12, BioLegend), CCR9–Alexa Fluor 647 (CW-1.2, BioLegend), IL-2–APC (JES6-5H4, BioLegend), CD45–APC-eFluor780 (30-F11, Invitrogen), and CD4–APC-eFluor780 (RM4-5, Invitrogen).

### IEL isolation using the conventional Percoll gradient method.

For IEL isolation using Percoll gradients (GE Healthcare), the SI was removed, flushed twice with cold 2% FCS in HBSS (Thermo Fisher Scientific), cut lengthwise to open the tube, and shaken twice in cold 2% FCS (HyClone) in HBSS. The opened SI tissues were then cut into 1 cm pieces and shaken twice with cold 2% FCS in HBSS. Afterward, the SI pieces were transferred into 37°C prewarmed solution A (5 mM EDTA [Quality Biologicals], 0.1 mM dithiothreitol [Sigma-Aldrich], and 10% FCS in HBSS) and shaken in conical flasks at 245 rpm at 37°C. After 45 minutes, the suspension was filtered through 70 μm Falcon filters and spun at 1500 rpm for 7 minutes. Pelleted cells were washed with cold PBS at 525*g* for 7 minutes at 10°C. A Percoll gradient of 80% Percoll and 40% Percoll was freshly prepared for each isolation. Cells were resuspended in 1 mL 40% Percoll and 1 mL Solution B (5 mM EDTA and 5% FCS in RPMI-1640 medium), layered on top of the Percoll gradient, and centrifuged at 935*g* for 25 minutes at 25°C. After 25 minutes, the top phase was discarded, and IELs were collected at the interphase between the 40% and 80% Percoll layers. The IELs were washed once with serum-free RPMI-1640 medium at 1500 rpm for 7 minutes before further analysis.

### IEL isolation by depletion of EpCAM^+^ epithelial cells.

For IEL isolation using anti-EpCAM antibodies (clone G8.8, Invitrogen), the SI was treated as described for Percoll gradient isolation up to the filtration through 70-μm Falcon filters and spinning the cell suspension at 525*g* for 7 minutes at 10°C. Afterward, the cells were washed with ice-cold PBS and resuspended in 10% FCS in HBSS at 20 million cells/mL. After adding purified anti-EpCAM antibodies (clone G8.8; 10 μL/mL), the cells were incubated on ice for 30 minutes, and excess antibodies were washed out with 10% FCS in HBSS. EpCAM^+^ cells were depleted by incubation with goat anti–mouse IgG BioMag beads (Qiagen) and goat anti–rat IgG BioMag beads (Qiagen).

### Cytokine production assessment.

IELs were cultured with 1 μg/mL plate-bound anti-CD3 antibody (145-2C11; BioLegend) in the presence of 10 ng/mL IL-2 (202-IL-500/CF, R&D Systems), 20 ng/mL IL-7 (407-ML-025/CF, R&D Systems), and 100 ng/mL IL-15 (447-ML-010/CF, R&D Systems). After 2 days, fresh IL-2 was supplemented (10 ng/mL), and the cells were cultured for an additional 3 days. To assess cytokine production, activated cells were harvested and stimulated with PMA (50 ng/mL; Sigma-Aldrich) and ionomycin (1 mM; Sigma-Aldrich) for 45 minutes, followed by brefeldin A (3 μg/mL; Invitrogen) treatment for 3 hours. Intracellular staining for TNF-α and IFN-γ was performed after fixation and permeabilization using an IC fixation kit (eBioscience), and cells were analyzed by flow cytometry. To assess IL-10 and IL-2 production, freshly isolated IELs and LN cells were stimulated with PMA (50 ng/mL; Sigma-Aldrich) and ionomycin (1 mM; Sigma-Aldrich) for 45 minutes, followed by brefeldin A (3 μg/mL; Invitrogen) treatment for 3 hours. Intracellular staining for IL-10 and IL-2 was performed after fixation and permeabilization of the stimulated cells using an IC fixation kit (eBioscience), and cells were analyzed by flow cytometry.

### Quantification of intracellular pSTAT5 contents in Foxp3^+^ cells.

Intracellular staining for pSTAT5 was performed using a previously described protocol (20). In brief, freshly isolated lymphocytes from the LN or SI epithelium were processed into a single cell suspension, and cells were fixed using a Foxp3 intracellular staining kit (eBioscience). The cells were then washed with permeabilization buffer (eBioscience) and subsequently stained with eFluor660-conjugated anti-Foxp3 antibodies for 30 minutes at room temperature. Afterward, the cells were washed, refixed using 2% paraformaldehyde at 4°C, and permeabilized using ice-cold 90% methanol. After 30 minutes on ice, excess reagents were washed out, and the cells were stained with anti-pSTAT5 antibodies at room temperature for 40 minutes, followed by staining with antibodies against the surface markers CD4 (GK1.5, Tonbo Bioscience), TCRβ (H57-597, BioLegend), CD8α (53-6.7, BD Biosciences), CD25 (PC61.5, eBioscience), and CD45 (30-F11, Invitrogen) for 20 minutes at room temperature. Afterward, the cells were washed and kept on ice until analysis by flow cytometry. For in vitro IL-2 stimulation, single cell suspensions were prepared from LN or EpCAM^+^-depleted SI IELs and incubated with recombinant IL-2 (1 ng/mL) for 30 minutes in a 37°C water bath. IL-2 stimulation was terminated by adding Foxp3 intracellular staining solution (eBiosciences), followed by washing with permeabilization buffer (eBiosciences). The intracellular pSTAT5 staining was performed as described above for freshly isolated cells.

### In vitro Foxp3^+^ Treg differentiation.

Naive CD4^+^ T cells sorted by FACS from *Foxp3*-GFP mice were induced to differentiate into Foxp3^+^CD25^+^ Tregs in vitro as previously described (35, 36). In brief, LN cells from *Foxp3*-GFP mice were sorted to isolate naive CD4^+^ T cells based on the surface markers CD4, CD44, and TCRβ. The purified T cells were then stimulated with 1 μg/mL plate-bound anti-CD3/CD28 antibodies (2C11/ 37.51; BioLegend) for 4 days in the presence of 5 ng/mL TGF-β (240-B-010/CF, R&D Systems), 10 ng/mL IL-2 (R&D Systems), and 10 μg/mL each of anti–IFN-γ (R4-6A2; BioLegend) and anti–IL-4 (11B11, BioLegend) antibodies. After the in vitro differentiation, cells were harvested, counted, and assessed for CD25 and *Foxp3*-GFP reporter protein expression by flow cytometry.

### Foxp3^+^ Treg suppression assay.

CD4^+^*Foxp3*-GFP^+^ cells were sorted by FACS from LN and IEL populations of *Foxp3*-GFP mice. Congenic (CD45.1) naive CD4^+^ T cells labeled with Cell Trace Violet (CTV; Invitrogen) were used as Tresp. CTV-loaded CD4^+^ Tresp were cocultured with equal numbers of CD4^+^*Foxp3*-GFP^+^ cells from the LN or SI IEL in the presence of anti-CD3 antibodies (0.5 μg/mL; BioLegend) and 2 × 10^5^ irradiated (2000 rads) splenocytes, which were included as antigen-presenting cells. Suppression of CD4^+^ Tresp proliferation was determined by assessing CTV dilution in Tresp after 5 days of in vitro stimulation.

### Adoptive transfer of iTregs.

In vitro differentiated Foxp3^+^ iTregs (CD45.2) and naive CD4^+^ T cells from congenic mice (CD45.1) were mixed at a 1:4 ratio and injected retroorbitally into lymphopenic *Rag2*^–/–^ recipient mice. iTregs were generated from naive CD4^+^ T cells isolated from *Foxp3*-GFP reporter mice, and their mature phenotype was confirmed by flow cytometry before FACS and injection. A total of 10 million iTregs were injected per host mouse, and the donor cells were recovered 8 weeks after injection from the LN and the SI epithelium of the host mice for further analysis.

### Statistics.

Statistical analyses were performed using Prism 8.0 software (GraphPad Software Inc.). Comparisons between groups were analyzed using a paired, 2-tailed Student’s *t* test (2 groups) or 1-way ANOVA followed by Tukey’s multiple comparisons test (more than 2 groups). Data are presented as the mean ± SEM. *P* values of less than 0.05 were considered significant (*P < 0.05, **P < 0.01, ***P < 0.001).

### Study approval.

Animal experiments were reviewed and approved by the National Cancer Institute, Animal Care and Use Committee. All mice were cared for in accordance with NIH guidelines.

## Author contributions

PP designed and performed the experiments, analyzed the data, and contributed to the writing of the manuscript. JADV, HK, AC, and XT performed the experiments, analyzed the data, and commented on the manuscript. YKP and JHP conceived the project, analyzed the data, and wrote the manuscript.

## Supplementary Material

Supplemental data

## Version 1 11/08/2021

Electronic publication
