# Supplementary material for: The small intestine epithelium exempts Foxp3+ Tregs from their IL-2 requirement for homeostasis and effector function
Source: JCI Insight. 2021 Nov 8;6(21):e149656. doi: 10.1172/jci.insight.149656 (PMC8663555; doi:10.1172/jci.insight.149656)
Supplement: Supplemental data [file jciinsight-6-149656-s104.pdf]

Supplementary Figure 1

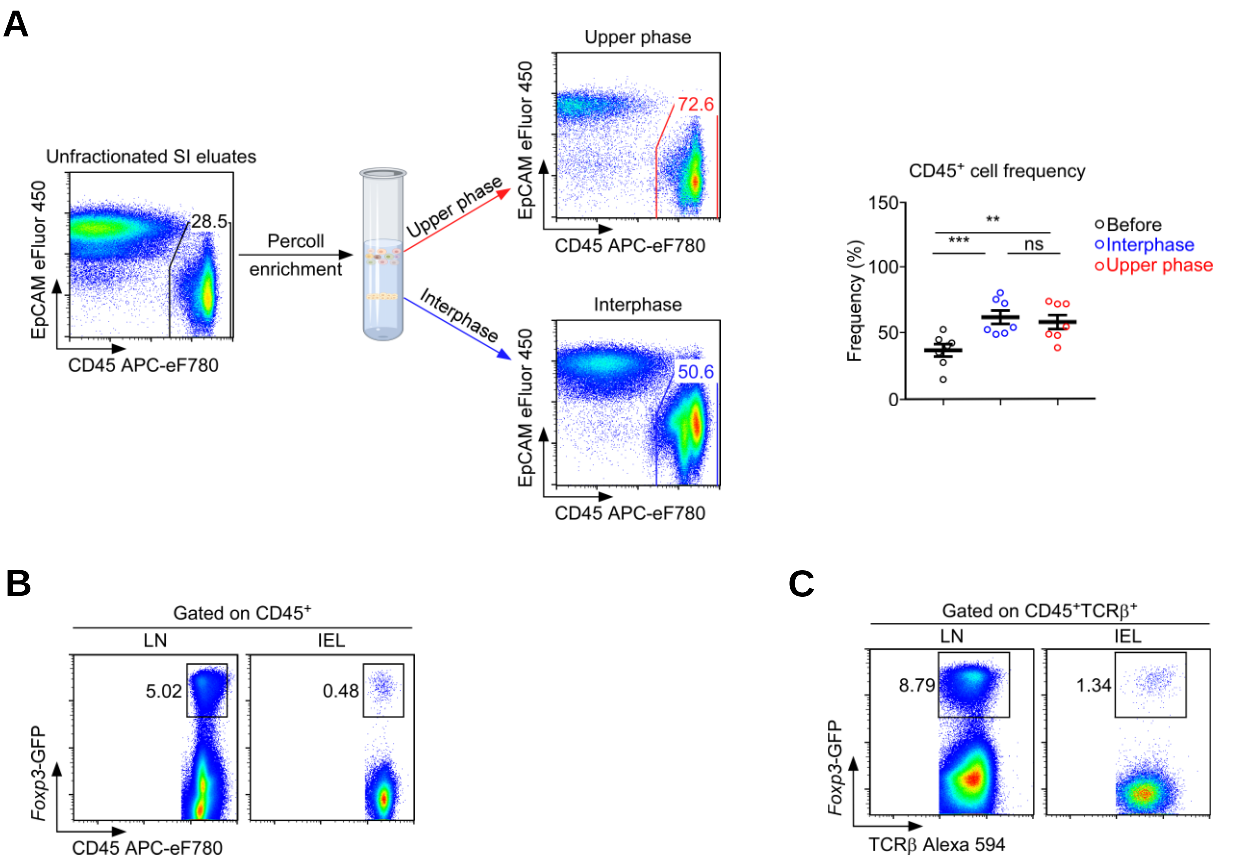

Suppl. Fig. 1 Percoll gradient method and Foxp3⁺ Treg cell frequencies among CD45⁺ IELs
A. EpCAM versus CD45 profile of SI IEL before Percoll enrichment (left), and for upper phase and interphase phases after Percoll enrichment (middle). Graph shows the CD45⁺ cell frequency as a summary of 3 independent experiments with a total of 7 WT mice (right).
B. Frequency of Foxp3⁺ Treg cells was determined among CD45⁺ LN cells and SI IEL that were enriched by the conventional Percoll gradient method from *Foxp3*-GFP reporter mice. Result is representative of 2 independent experiments with a total of 4 *Foxp3*-GFP reporter mice.
C. Frequency of Foxp3⁺ Treg cells was determined among CD45⁺TCRβ⁺ LN cells and SI IELs that were enriched by the conventional Percoll gradient method from *Foxp3*-GFP reporter mice. Result is representative of 2 independent experiments with a total of 4 *Foxp3*-GFP reporter mice. Data are represented as mean ± SEM. *P* values were determined by one-way ANOVA with Tukey's multiple comparison test . **, $P < 0.01$; ***, $P < 0.001$; ns, non-significant.

Supplementary Figure 2

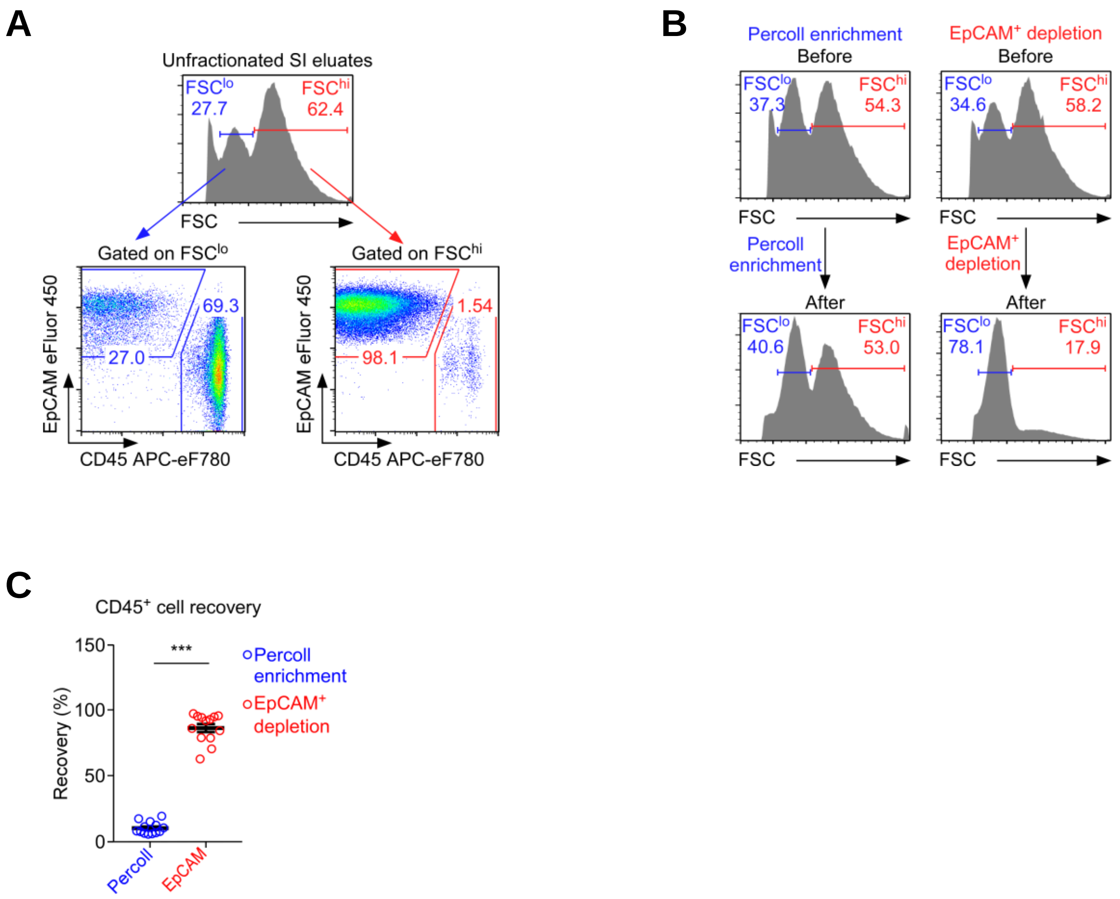

Suppl. Fig. 2 IEL isolation by EpCAM⁺ cell depletion

A. Forward scatter (FSC) analysis of SI IELs. FSC^{lo} and FSC^{hi} cells were assessed for EpCAM and CD45 expression. The $EpCAM^{+}CD45^{-}$ gate corresponds to epithelial cells, while the $EpCAM^{-}$ $CD45^{+}$ gate identifies cells of hematopoietic origin. The data are representative of 9 independent experiments with a total of 13 WT mice.

B. Forward scatter (FSC) analysis of SI IEL before and after Percoll enrichment (left) or $EpCAM^{+}$ depletion (right). The results are representative of 9 experiments.

C. Recovery of $CD45^{+}$ SI IELs from WT mice after Percoll enrichment or $EpCAM^{+}$ cell depletion. The percentage recovery was determined by dividing the number of $CD45^{+}$ cells after $EpCAM^{+}$ depletion by the number of $CD45^{+}$ cells in the SI epithelial lavage before depletion and then multiplying by 100. The graph shows the summary of 9 independent experiments with a total of 13 WT mice. The data are represented as the mean ± SEM. *P* values were determined by paired Student's *t*-test. $^{***}P < 0.001$.

Supplementary Figure 3

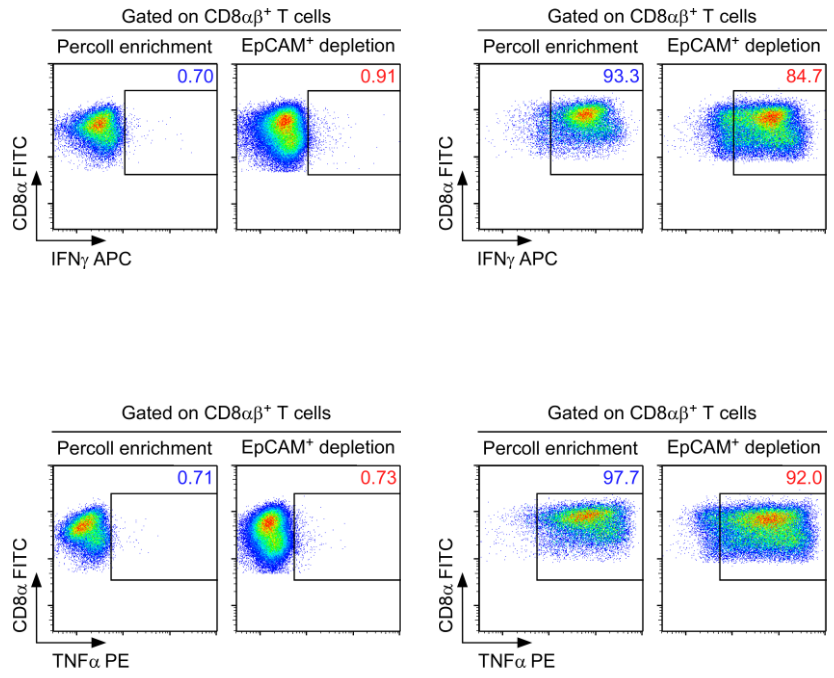

Suppl. Fig. 3 Comparison of IELs isolated by Percoll gradients and EpCAM$^+$ depletion
Intracellular staining of IFNγ (top) and TNFα (bottom) in CD8αβ$^+$ T cells that were isolated from SI epithelial tissues by either Percoll enrichment or EpCAM$^+$ depletion and then TCR stimulated for 5 days *in vitro*. Contour plots show cytokine expression before (left) or after (right) stimulation with PMA and ionomycin for 4 hours. The results are representative of 2 independent experiments with a total of 4 WT mice.

Supplementary Figure 4

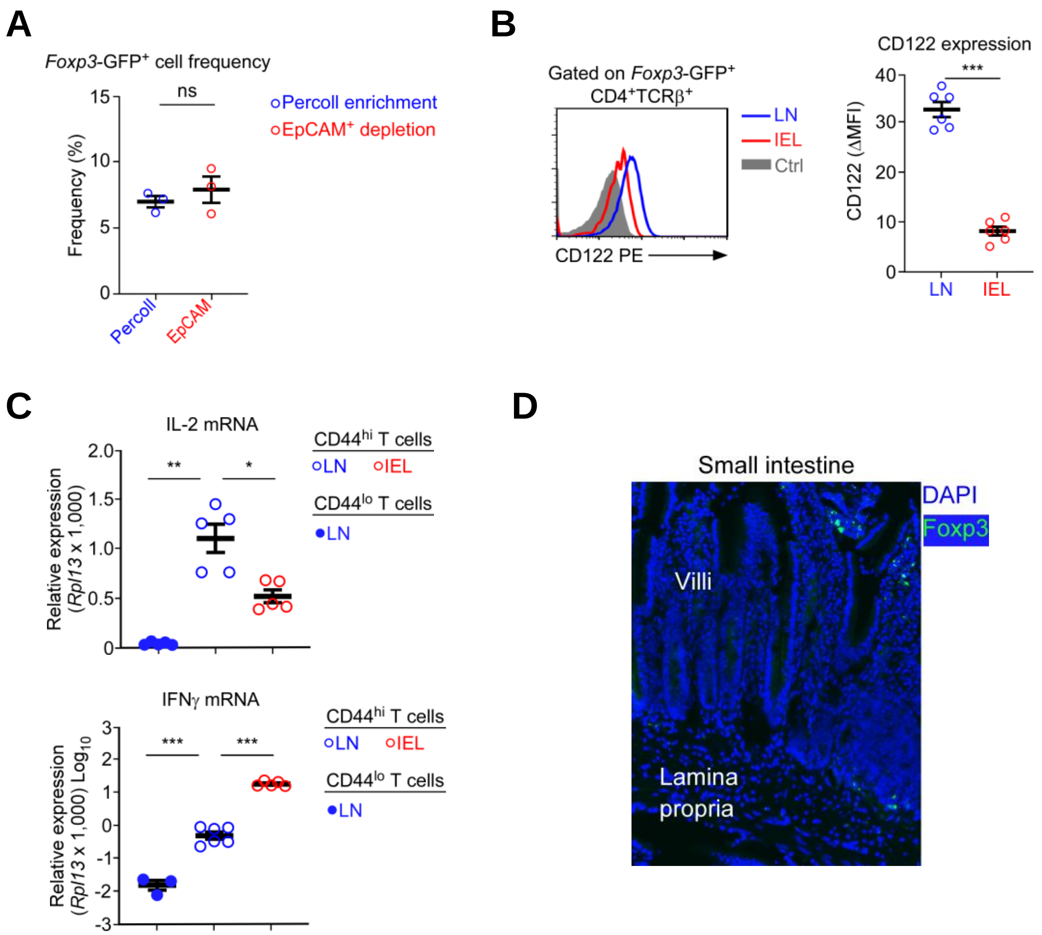

Suppl. Fig. 4 Phenotypic analysis of SI IEL Foxp3$^+$ Treg cells

A. Frequencies of *Foxp3*-GFP$^+$ T cells among CD45$^+$ IELs that were isolated either by Percoll enrichment or EpCAM$^+$ deletion. Graphs show the summary of 2 independent experiments with a total of 4 *Foxp3*-GFP reporter mice.

B. Histogram shows CD122 expression on *Foxp3*-GFP$^+$CD4$^+$TCRβ$^+$ cells of LN and IELs. Graph shows ΔMFI of surface CD122 expression. Histogram is representative and the graph is the summary of 2 independent experiments with a total of 6 *Foxp3*-GFP reporter mice.

C. Quantitative RT-PCR analysis of IL-2 and IFNγ mRNA expression in CD44^{hi} T cells from LN and IEL or from CD44^{lo} LN T cells. The relative abundance of cytokine mRNA was normalized to control *Rpl13* mRNA. The graphs show the summary of 2 independent experiments with a total of 3-6 WT mice.

D. Confocal microscopic image of Foxp3$^+$ cells (green) in the small intestine epithelium. Foxp3$^+$ Treg cells were identified with anti-Foxp3 antibodies followed by fluorescence-conjugated secondary antibodies, and the tissue sections were counterstained with DAPI (blue).

Data are represented as mean ± SEM. *P* values were determined by paired Student's *t*-test (A and B) or one-way ANOVA with Tukey's multiple comparison test (C). *, $P < 0.05$; **, $P < 0.01$; ***, $P < 0.001$; ns, non-significant.

Supplementary Figure 5

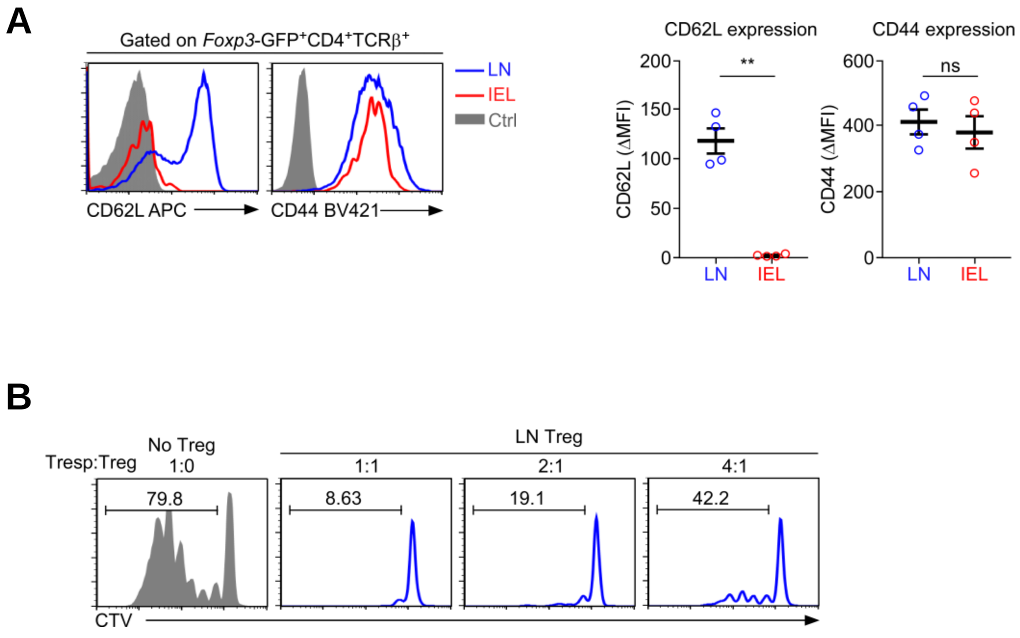

Suppl. Fig. 5 Functional analysis of Foxp3$^+$ Treg cells among SI IELs

A. Histograms show CD62L and CD44 expression on *Foxp3*-GFP$^+$CD4$^+$TCRβ$^+$ cells of LN and IELs (left). Graphs show the ΔMFI of CD62L and CD44 expression (right). Histograms are representative and the graphs show a summary from 2 independent experiments with a total of 4 *Foxp3*-GFP reporter mice.

B. Suppression assay using LN Tregs isolated from *Foxp3*-GFP reporter mice. Tresp proliferation was assessed after 5 days of culture with different ratios of Tregs cells. The data are represented as mean ± SEM. *P* values were determined by paired Student's *t*-test. **, *P*<0.01; and ns; non-significant.

Supplementary Figure 6

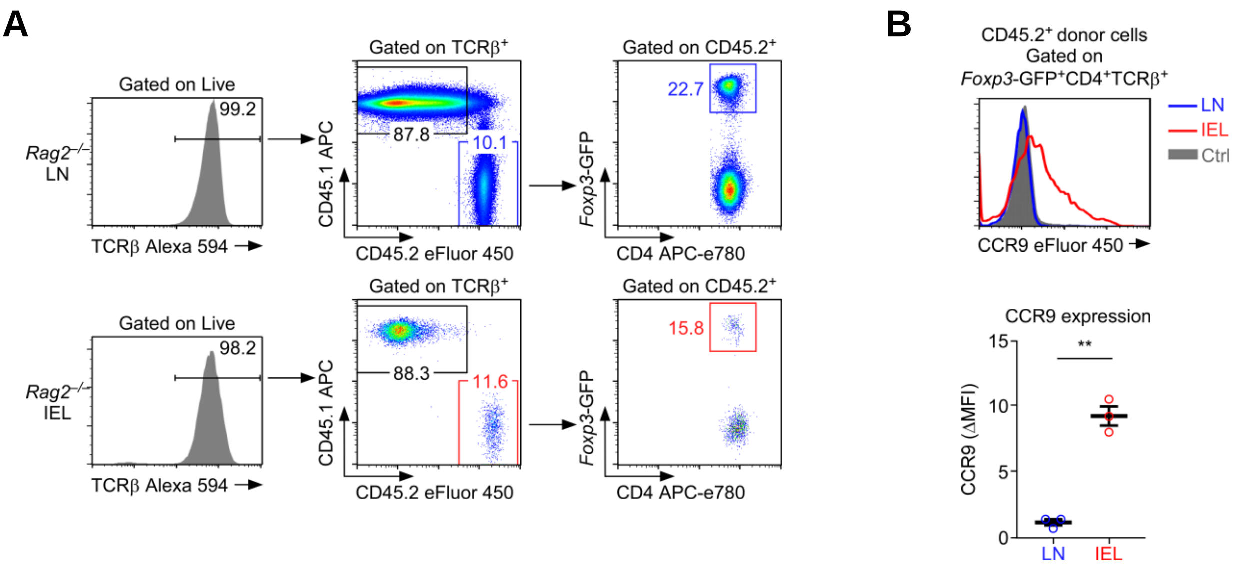

Suppl. Fig.6 Adoptive transfer and analysis of iTreg cells

A. Identification of donor iTreg cells in LN and IEL of *Rag2*$^{-/-}$ mice. Live donor cells were gated for TCRβ^{+} cells (left) followed by congenic markers CD45.1 and CD45.2 (middle). CD45.2^{+} iTreg were identified by *Foxp3*-GFP expression in CD4^{+} cells (right) and further analyzed. Results are representative of 3 independent experiments with a total of 3 *Rag2*$^{-/-}$ mice.

B. Surface staining for CCR9 in *Foxp3*-GFP^{+}CD4^{+}TCRβ^{+} donor T cells from LN and IELs of *Rag2*$^{-/-}$ recipient mice. The histogram is representative, and the graph shows the summary of 3 adoptive transfer experiments. The data are represented as mean ± SEM. *P* values were determined by paired Student's *t*-test. **, *P*<0.01.

Supplementary Methods

Quantitative RT-PCR.

Total RNA was isolated from sorted cells with RNeasy Mini kit (Qiagen). RNA was reverse transcribed into cDNA by oligo(dT) priming with the QuantiTect Reverse Transcription kit (Qiagen). A QuantStudio 6 Flex Sequence Detection System and the QuantiTect SYBR Green detection system (Qiagen) were used for quantitative RT-PCR. Primers sequences are as follows. IL-2 (forward, 5′-CCGCTCCCACTCCGATTA-3′, and reverse,5′-GCACCAAGAAGGTGCCA-3′), IFNγ (forward, 5′-CAGCAACAGCAAGGCGAAAAAGG-3′, and reverse, 5′-TTTCCGCTT CCTGAGGCTGGAT-3′) and Rpl13 (forward, 5′-CGAGGCATGCTGCCCCAC AA-3′, and reverse, 5′-AGCAGGGACCACCATCCGCT-3′).

Immunofluorescence microscopy

Mouse small intestine tissues were collected, washed, and opened longitudinally. The tissues were embedded in Tissue-Tek O.C.T. compound (SAKURA Finetechnical Company) in cryomolds, and snap frozen in liquid nitrogen for cryosectioning. Cryosections were prepared on a Leica Cryostat (Leica Microsystems) at −21°C in 5 μm thickness. Sections were mounted on glass slides and fixed in 100% ethanol at 4°C for 30 min followed by 3 min of −20°C acetone fixation at room temperature. The tissue sections were blocked in 5% donkey serum (Jackson laboratory) and 1% TritonX-100 in PBS. The monoclonal antibody anti-Foxp3 (Life Technologies) and Alexa 488 conjugated donkey anti-rat secondary antibody (Life Technologies) were used to detect Foxp3 location and DAPI was used to stain nucleus.
